# The Effects of Steroid Implant and Dietary Soybean Hulls on Estrogenic Activity of Sera of Steers Grazing Toxic Endophyte-Infected Tall Fescue Pasture

**DOI:** 10.3389/fvets.2015.00030

**Published:** 2015-09-04

**Authors:** Nancy W. Shappell, Michael D. Flythe, Glen E. Aiken

**Affiliations:** ^1^USDA-ARS Animal Metabolism-Agricultural Chemicals Research, Fargo, ND, USA; ^2^USDA-ARS Forage-Animal Production Research Unit, Lexington, KY, USA

**Keywords:** phytoestrogens, isoflavones, fescue toxicosis, prolactin, estrogenic activity

## Abstract

Soybean hulls (SBHs) have been fed to cattle pasturing on endophyte-infected tall fescue in attempts to increase rate of gain. Literature reports indicated some symptoms associated with fescue toxicosis were ameliorated by the use of steroidal implants containing estradiol (E2) and progesterone [implantation (IMP)], feeding SBHs, or the combination of the two. While the mechanism for amelioration was unclear, the SBHs were postulated as acting as a diluent of the toxic factors of the fescue. Alternatively, estradiol and phytoestrogens of SBHs might be acting through relaxation of the persistent vasoconstriction found in animals ingesting ergot alkaloids of endophyte-infected fescue. If so, estrogenic activity of serum of steers receiving SBHs, IMP, or a combination of the two should be elevated. Using the cellular proliferation assay of estrogenicity (E-Screen), estradiol equivalents (E_2_Eqs) were determined on both SBHs and the serum of steers from a previously reported study. Range of SBHs was 5.0–8.5 ng Eqs g^−1^ DM (mean 6.5, *n* = 4 from different commercial sources of SBHs). At the rate fed, theoretically calculated blood E_2_Eq could be physiologically relevant (~80 pg mL^−1^, based on 2.3 kg SBHs d^−1^, 300 kg steer, 5.7% blood volume, and 10% absorption). Serum E_2_Eqs did increase in steers (*P* ≤ 0.05) with steroidal implants or fed SBHs by 56 and 151% over control, respectively, and treatments were additive (211% increase). Serum prolactin was also greatest for the SBH + IMP group (188 ng mL^−1^, *P* < 0.05), concentrations comparable to values reported for steers grazing endophyte-*free* fescue. Prolactin in the SBH group was higher than IMP or control groups (146 versus 76 and 60 ng mL^−1^, respectively). Still unknown is if additional E_2_Eqs from dietary phytoestrogens or exogenous sources of estradiol can further reduce symptoms of fescue toxicosis. The E-Screen assay was an effective tool in monitoring serum for estrogenic effects of dietary supplementation with SBHs or estrogenic implants.

## Introduction

Ergot alkaloids produced by a fungal endophyte [*Epichloë coenophiala* (Morgan-Jones & W. Gams) C.W. Bacon & Schardl] that infects tall fescue [*Lolium arundinaceum* (Schreb.) Darbysh] have a strong affinity for selected subclasses of α-adrenergic and serotonin receptors in the vasculature (1, 2). Ergot alkaloids consumed by grazing animals cause persistent constriction of blood flow to peripheral tissues, thereby disabling the animal’s ability to regulate core body temperature, resulting in a condition referred to as fescue toxicosis. Various feeding regimens have been evaluated to mitigate the effects of fescue toxicosis on the rate of gain of steers (3–7). Soybean hulls (SBHs) are a high-fiber feed, the feeding of which has been effective in providing a higher plane of nutrition, resulting in a higher rate of gain, when fed to steers grazing toxic tall fescue pasture (6, 7). Feeding SBHs at a daily rate of 2.3 kg day^−1^ steer^−1^ partially reversed the decreased serum prolactin concentrations and decreased the frequency of rough hair coats [Carter et al. (7)], both symptoms of fescue toxicosis (8). An estradiol–progesterone implant with no SBH treatment also reduced prevalence of rough hair, and a combination of the two treatments resulted in an additive effect on mitigating toxicosis. While the authors conjectured that SBHs diluted ergot alkaloids in steer diets, the phytoestrogens in SBHs could have been a contributing factor in the mitigation through positive effects on specific vasculature and overall circulation.

Though phytoestrogens (a subset known as isoflavones) have been linked to reproductive issues in cattle and sheep (9, 10), dietary isoflavones have also been demonstrated to benefit human health and well-being (11, 12). Various effects on the vasculature have been reported. For instance, isoflavones have been identified as an alternative to 17β-estradiol in ameliorating hot flashes in post-menopausal women, through relaxation of the vasculature by binding the estrogen receptors in the vascular endothelium, stimulating nitric oxide synthase (NOS), resulting in nitric oxide-mediated vasodilation (12–14). There also is evidence that genistein, considered the most estrogenic phytoestrogen in soybeans, exerts a vasodilatory effect as a β-adrenergic receptor agonist (15). Based on these findings, isoflavones may have the potential to induce relaxation of alkaloid-induced vasoconstriction, and perhaps mitigate other signs of toxicosis as described by Carter et al. (7) (rough hair coat, low prolactin concentrations, and poor thriftiness/performance).

Depending on species, variety, season and drying time, the amount of isoflavones found in legumes, such as soybeans, alfalfa, and clover, can vary (11, 16–18). While the estrogenic activity of soybean meal (SBM) (45% crude protein) was assessed using the *in vitro* cellular proliferation assay (E-Screen) and found to average 113 ng g^−1^ as fed (19), the estrogenic activity of SBHs has not been evaluated. Therefore, SBH samples collected from various suppliers, and serum samples collected from steers of the Carter et al. (7) study were analyzed by E-Screen to assess the estrogenicity of SBHs and determine if there is an association between serum prolactin and serum estrogenic activity.

## Materials and Methods

### Soybean meal and hulls

Samples of SBHs fed by Carter et al. (7) were no longer available for determination of estrogenic activity, therefore four samples from unrelated lots of SBHs were obtained from three feed suppliers representing three different years of harvest (Table 1, Woodford Feed Co., Versailles, KY, USA; Hallway Feed Co., Lexington, KY, USA, and North Dakota State University Feed Mill, Fargo, ND, USA). Sample size ranged from 120 to 750 g (as fed). Sample dry matter was determined in quintuplicate (dried at 100°C). Extractions were done as described for isoflavinoid analysis (20), with the exception of an additional hexane extraction step. Three subsamples of feed (1 g as fed) were extracted using sonication (47 kHz) and 25 mL of 60% acetonitrile at 37°C for 1 h, shaking samples every 10 min to resuspend feed and enhance sample/solvent contact. Particulates were removed by centrifugation (900 × *g*, 10 min). Supernatants were filtered through solvent-washed glass wool to remove remaining fines and volumes recorded. Acetonitrile was volatilized under N_2_ at 37°C, and hexane (1:1, v:v) was added, vortexed for 1 min, then held at −20°C for ≥1 h. Phase separation was achieved by centrifugation as described above. The upper hexane layer was discarded, and the aqueous layer was subsequently diluted to ~90 mL with nanopure H_2_O (npH_2_O) after removing any pelleted material. Phytoestrogens were then concentrated by Oasis HLB solid phase extraction as described [Waters, Milford, MA, USA (21)], and the eluent taken to dryness and stored at −20°C for later analysis. On the day of assay, samples were resuspended in 400 μL of 5% dimethyl sulfoxide in npH_2_O. Resuspended SBH samples were diluted (1:10,000–1:25,000) in cell culture medium described below to obtain cellular responses in the linear range of the E-Screen assay (~1 × 10^−12^ to 1 × 10^−11^ M).

**Table 1:** **Estrogenic equivalents of commercial soybean hull feedstuff by E-Screen assay**.

Site of purchase/origin	Year of harvest	E_2_Eqs^a^ ng E_2_Eqs g^−1^ DM	COV^b^
KY/na^c^	2011	8.5 ± 0.7	8%
ND/na	2013	5.0 ± 0.7	14%
KY/IN	2014	7.2 ± 0.3	5%
KY/IN	2014	5.3 ± 0.4	8%
	Mean	6.5 ± 1.6	25%

*^a^Mean ± SD of three replicate extractions, each dilution was tested on 4–6 wells of cells. Values do not reflect activity assayed in hexane phase, as ≤3% of SPE values*.

*^b^Coefficient of variation*.

*^c^Not available*.

### Animal treatment, serum collection

Estradiol equivalents were assayed on serum collected from steers used in a 2-year grazing experiment, evaluating the effect of combinations of steroid implantation (IMP) and SBH feeding on mitigation of fescue toxicosis (7). The experimental protocol was reviewed and accepted by Institution’s Animal Care and Use Committee at the University of Kentucky (00996A2006). Treatments consisted of IMP (Synovex-S 200, 200 mg progesterone and 20 mg estradiol, Fort Dodge Animal Health, Fort Dodge, IA, USA), SBHs (daily SBH feeding at 2.3 kg steer^−1^ day^−1^), SBH + IMP (combined feeding of SBHs and IMP), and Control (pasture-only). Steers were assigned to 12 3.0-ha pastures of toxic endophyte-infected tall fescue in a split-plot design. Main- and sub-plot treatments were ±SBH and ±IMP. Blood was collected from the jugular vein on 14 June and 5 July in 2007, and 24 June and 24 July in 2008. Blood was centrifuged for 15 min at 10,000 × *g* to obtain serum, which was stored frozen (−20°C) for prolactin analysis. Detailed descriptions of experimental design, sample preparation, and analyses (including prolactin) were provided by Carter et al. (7). Estrogenic activity (estradiol equivalents, E_2_Eqs) was analyzed in serum from a subset of steers for which serum samples were available for all collection dates to provide an estimate of the bioavailability of isoflavones once they are absorbed into the animal’s circulation.

### Serum extraction and E-Screen

Prior to E-Screen testing, serum samples required extraction to eliminate factors toxic to the MCF-7 cells as previously described (22). Sterile aliquots (typically 1 mL) of serum were diluted with acetonitrile (ACN, 1:2 v/v) in silanized glass conical centrifuge tubes. Serum weights were recorded, samples vortexed for 1 min, and centrifuged at 800 × *g* for 10 min (room temperature). The serum/ACN was removed from the pellet and transferred into a silanized glass vial, and residual liquid and pellet were weighed. The extracted serum was taken to dryness, and stored at −20°C until E-Screen analysis. Sample weights were used to calculate and adjust final estrogenic activity values for sample loss during extraction. Extracted serum samples were reconstituted in npH_2_0 at 1/5th the original serum volume. Samples were further diluted in cell culture medium (1:10 to up to 1:25 of the original serum) to obtain a proliferative response in the linear range of the E_2_ standard curve. The limit of quantitation was ~0.2 pg mL^−1^ of E_2_Eq in the original serum.

The MCF-7 BOS, estrogen-dependent cell line (derived from a human mammary epithelial carcinoma, provided by Drs. Ana Soto and Carlos Sonnenschein, Tufts University, Boston, MA, USA) was used to determine estrogenicity relative to 17β-estradiol as described by Shappell (21). Briefly, resuspended extracts were diluted in cell culture media. Twenty-four hours post-plating, steroid-containing medium (5% FBS) was removed and replaced with steroid-free medium (minus phenol red and containing 10% charcoal dextran-stripped FBS) containing diluted sample extracts of samples or 17β-E_2_ (1 × 10^−13^ to 1 × 10^−9^ M). After 5 days of incubation, cells were fixed with trichloroacetic acid, stained for protein with sulforhodamine B (Sigma Chemicals, St. Louis, MO, USA), solubilized in buffer, and absorbance measured (490 nm). Estradiol equivalents were determined based on a regression analysis of the 17β-E_2_ curve from the same experiment. Assay assessment, including range finding, was performed on serum samples from four steers on endophyte-infected tall fescue pasture, but not on trial. All extracts were tested over a wide range of dilutions, and those resulting in absorbance readings in the linear range of the E_2_ standard curve were used for interpolation of the data. Evidence of toxicity was determined by evaluating cellular proliferation in the presence of the sample extract spiked with 17β-E_2_ versus 17β-E_2_ alone. The estrogen-dependence of cellular proliferation was confirmed by co-incubation with the E_2_-receptor antagonist ICI 182,780 [Tocris, Ellisville, MO, USA (23)].

### Statistical analysis

Estradiol equivalents and prolactin concentrations were statistically analyzed using mixed models of SAS (24), with year, treatment, and blood collection month (June versus July) as fixed effects, and animal as a random effect. Mean separations were performed on least square means using the Tukey–Kramer adjustment of multiple comparisons of least square means. Estradiol equivalents data were transformed using the log function to stabilize the variances and analyzed as repeated measures using the AR(1) covariance structure.

## Results

### Soybean hull estrogenic activity

Quantitation of E_2_Eqs from extracts of SBHs yielded reproducible data. Coefficient of variation on triplicate extractions averaged 8.8%, with only one COV over 10% (14.2%). The hexane extractable phase contained ≤3% of the estrogenic activity from the SPE extraction. Estradiol equivalents of the SBH samples harvested in three different years and representing at least two different regions averaged 6.5 ± 1.65 ng g^−1^ dry matter or 5.8 ng g^−1^ wet weight (Table 1, overall COV 25%). As expected, the dry matter content of the processed SBHs were essentially identical (89 ± 1%). The estrogenic activity of SBH samples was more variable, though the range of values within a year was similar to the range across years.

### Serum E_2_Eqs

**Figure 1:**
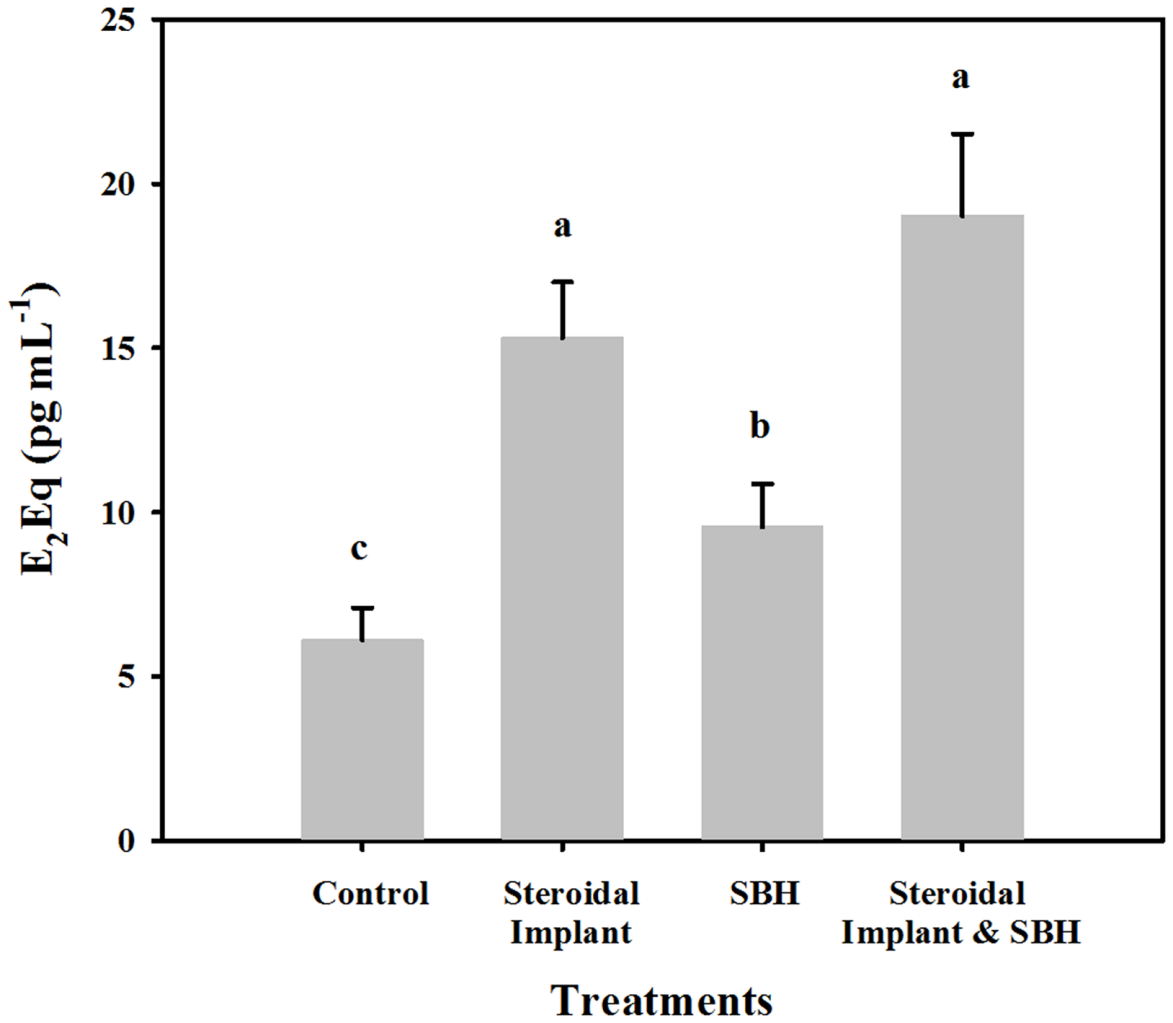
**Mean estrogenic equivalents (E_2_Eqs) in steers grazing toxic endophyte-infected [*Epichloë coenophiala* (Morgan-Jones & W. Gams) C. W. Bacon & Schardl] tall fescue [*Lolium arundinaceum* (Schreb.) Darbysh] and treated (*n* = 4 steers per treatment groups) with or without an estradiol–progesterone ear implant and soybean hull (SBH) feeding combinations**. Values are averages over 2 years, two sample dates per year (6/14 and 7/5/2007; 6/24 and 7/24/2008). Means with different letters are different (*P* ≤ 0.05).

Range finding samples from the four steers on endophyte-infected tall fescue pasture, but not on trial yielded values similar to those from the control treatment group (4.9 ± 0.92 versus 6.1 ± 1.0 pg mL^−1^ E_2_Eqs, respectively). Of the experimental treatments, all had mean serum estrogenicity greater (*P* < 0.05) than control steers (Figure 1). The smallest increase in E_2_Eqs over control was found when feeding SBHs alone (9.6 ± 1.3 pg mL^−1^), with the serum from IMP steers ranking next (15.3 ± 1.7 pg mL^−1^). The highest mean serum E_2_Eqs was of steers receiving the combined IMP and SBH treatment (19.0 ± 2.5 pg mL^−1^ and similar to IMP alone (*P* = 0.44). Serum E_2_Eqs of IMP-SBH steers was greater (*P* < 0.001) than the SBH steers, indicating an additive effect of the two treatments.

### Serum prolactin

**Figure 2:**
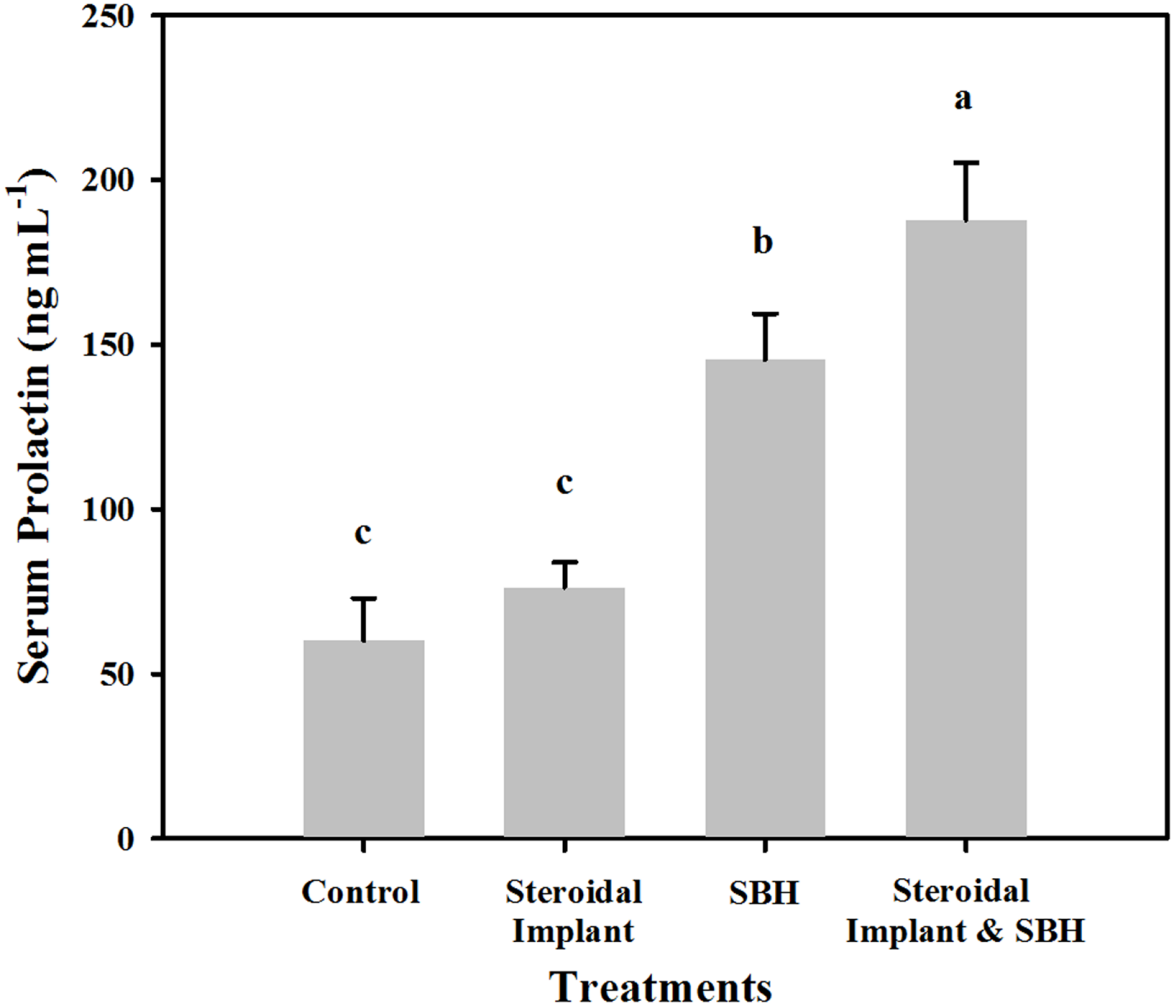
**Mean serum prolactin levels in steers grazing toxic endophyte-infected [*Epichloë coenophiala* (Morgan-Jones & W. Gams) C. W. Bacon & Schardl] tall fescue [*Lolium arundinaceum* (Schreb.) Darbysh] and treated (*n* = 4 steers per treatment groups) with or without an estradiol–progesterone ear implant and soybean hull (SBH) feeding combinations**. Values are averages over 2 years, two sample dates per year (6/14 and 7/5/2007; 6/24 and 7/24/2008). Means with different letters are different (*P* ≤ 0.05).

Unlike serum estrogenic activity, serum prolactin concentrations of IMP steers were not different from control concentrations (76.3 ± 7.6 IMP, versus 60.1 ± 12.9 ng mL^−1^ control, *P* = 0.38). SBH treatment alone resulted in elevated prolactin concentrations (145.5 ± 13.9 ng mL^−1^, *P* < 0.05) with even greater prolactin concentrations when SBH and IMP treatments were combined (187.9 ± 17.3 ng mL^−1^, *P* < 0.05) (Figure 2). There was a weak, but positive correlation between serum E_2_Eqs and serum prolactin concentrations (*r* = 0.29; *P* = 0.027; *n* = 59).

## Discussion

### Estrogenic activity of SBHs

While soybeans and SBM have been evaluated for phytoestrogens and estrogenic activity, SBHs as an alternative feedstuff had not. The hulls are a co-product of milling meal that is derived from the fibrous, inert, soybean seed coat and are greater than 86% cellulose, hemicellulose, and lignin (25), with the remainder being primarily protein and minerals. Variability of E_2_Eqs (5.0–8.5 ng g^−1^) reported herein for SBHs from different suppliers was less than that reported for soybean *meal* using the yeast-based human α-estrogen receptor *in vitro* assay [YES (26)]. Meal from lots manufactured 1 month apart varied threefold in E_2_Eqs by YES assay, while SBHs analyzed here varied by <2-fold. For context, based on data from our laboratory, the estrogenic activity of SBHs could be expected to range from 4 to 20% of SBM. These estimates are based on E-Screen analysis of SBM in our laboratory yielding 127 E_2_Eqs ng g^−1^ dry matter (19), and are similar to analysis by YES assay [29–100 E_2_Eqs]. The measured quantities of estrogenic activity of SBH are in line with the phytoestrogen content of hulls, reported to be 11–15% of whole soybeans or 15% of SBM from chemical analysis (27).

### Physiological context for estrogenic activity

A theoretical assessment of the potential for a physiological effect of SBH feeding of steers follows:

2,070 g DM SBHs per day (2,300 g as fed × 89% dry matter)
2,070 g SBHs DM × 6.5 E_2_Eqs ng g^−1^ DM SBHs = 13.5 μg E_2_Eqs per day per steer
For a 300 kg steer, blood volume estimates = 5.7% of body weight (28)
300 kg × 5.7% = 17.1 L or 17,100 mL blood volume
Of 13.5 μg E_2_Eqs fed, if 10% are absorbed and bioavailable, then 1.35 μg E_2_Eqs/17,100 mL blood = 79 pg mL^−1^ E_2_Eqs in blood.

Obviously, this estimate has inherent assumptions requiring caution, if not skepticism. First, is the assumption of bolus delivery of phytoestrogens, second, no metabolism of phytoestrogens, either by steer or by their microflora, reducing the estrogenicity of the feedstuff. Though ingested phytoestrogens are found circulating in the serum of cattle (29), estimates of bioavailability were not reported. The peak blood concentrations (relative to time of consumption) resembled those in a study of women consuming phytoestrogen-rich diets (30). In women, the estimates of bioavailability for two phytoestrogens (genistein and daidzein) were 18–27% based on urinary recovery after 48 h. Similarly, oral bioavailability of genistein and daidzein in rats were 18 and 23%, respectively (31). Using a value of 10% bioavailability in the calculation above, though less than these reports, could still result in large overestimates due to ruminal metabolism (32). The estimate above also ignores any receptor-level competition between naturally circulating estrogens and the phytoestrogens. For context, the circulating concentration of estradiol for cows in estrus peak at ~10 pg mL^−1^ (28). Based on this calculation, it appears the dosage of estrogenic activity from SBHs, may be capable of eliciting an estrogenic response.

Supporting the potential for circulating E_2_Eqs of steers consuming SBHs to be capable of exerting a physiological impact are the findings in rats receiving phytoestrogens. After 3 days of oral administration of ~75 mg kg body weight^−1^ day^−1^ of the phytoestrogens, genistein and daidzein (2:1 ratio), to 17 day old rats, an increase in wet uterine weight was observed (33). SBHs (as fed) were reported to contain 111 mg kg^−1^ of genistein and 200 mg kg^−1^ of daidzein (27). Using these values, the steers in our study received an estimated 250 and 460 mg kg body weight^−1^ day^−1^ of genistein and daidzein, respectively, substantially more than the above threshold for a uterine dose response in rats.

### Serum E_2_Eqs and prolactin response to treatment

Historically little attention has been paid to the estrogenicity of various livestock feeds in comparison to estrogenicity of human foods. Exceptions include red clover and the associated infertility in sheep (34) or the effects of the estrogenic mycotoxin zearalenone-contaminated feed on heifers (35) and ewes (36). Soybeans have been used for livestock and poultry feed in the United States since the 1930s (37), and SBM is referred to as “the most important protein source used to feed farm animals” by the Feedipedia web site sponsored by the Institute for Agricultural Research, French Agricultural Center for International Development, French Association for Animal Production and the Food and Agriculture Organization of the United Nations (38). In contrast, their web site which provides many pages of information on SBM, mentions SBM’s estrogenic properties in only one line – “soybean meal contains 1 g kg^−1^ of genistein, which has estrogenic properties.” In contrast, a recent review in the International Journal of Endocrinology reports on the effect of phytoestrogens and specifically SBM on reproductive hormones in cows (29). SBHs, which contain only 5–20% of the estimated E_2_Eqs of commonly fed SBM, nearly doubled the serum E_2_Eq concentration of steer serum relative to control. These findings, at least conceptually, support the theoretical estimates of estrogenic activity made above, and are further supported by empirical findings of increased estrogenic activity of serum from SBH + IMP steers.

Estrogenic activity of serum from implanted steers was almost threefold that of control steers. These findings are in agreement with the plasma E_2_ concentrations measured in steers with Synovex-S implants (20 mg estradiol). In two separate trials, Rumsey and Beaudry (39) found mean E_2_ concentrations to be 4.4 and 34 times the non-implanted steers concentration, 60 days post-implantation (*n* = 10 steer per treatment per trial, using radioimmunoassay), with mean E_2_ concentrations declining to near control concentrations by 106–120 days. Estrogenic activities in serum samples from steers in the current study are a mean of those collected at about 60 and 90 days post-implantation, and therefore could be expected to be lower than at 60 days.

While the findings of enhanced estrogenic activity with SBH feeding is novel, perhaps more unexpected is the trend for additivity of treatments (SBH + IMP) on E_2_Eqs. The utility of the E-Screen assay can be fully realized here, because measurement of blood estrogens alone, either by immunoassay or by chemical analysis, would have failed to accurately reflect the full potential for systemic estrogenic activity. There have been some indications of additivity of *in vivo* responses to estrogenic chemicals in the literature, though often evidence of additivity occurs over a very limited range of concentrations [fish: (40, 41); frogs: (42); rats: (33)]. Lack of additive responses are most likely a reflection of the complexity of nature including differential rates of absorption, metabolism, receptor affinity, biofeedback, etc. The E-Screen assay has the unique potential to evaluate the effect of estrogenic modulators mid-experiment for an indication of an *overall* change in estrogenic activity of the animal’s serum, not a specific hormonal response. Results from such monitoring could lead investigators to end experiments earlier than anticipated, or extend them.

One of the few measurable parameters indicative of fescue toxicosis is a decreased serum prolactin concentration (8). The control steers grazing endophyte-infected fescue had serum prolactin concentrations similar to bulls fed endophyte-infected fescue seed [~30 ng mL^−1^ (43)], indicating toxicity. While implants caused no significant increase in serum prolactin, SBHs did elevate prolactin, and the combination of SBH + IMP resulted in prolactin concentrations similar to those of bulls fed endophyte-free fescue seed [~250 ng mL^−1^ (43)]. In other words, the ~3-fold decrease in prolactin concentrations measured with consumption of endophyte-infected fescue, was reversed in SBH + IMP steers. The lack of elevation in serum prolactin in the IMP steers is puzzling, but could reflect suppression by progesterone contained in the implants. The literature has no reports of progesterone effects on prolactin in steers. Implants of estradiol alone are not widely used, but it would be of interest to compare serum prolactin in steers implanted with E2 versus E2 plus progesterone. These results appear to indicate that phytoestrogens of SBHs could be acting as surrogates for estradiol, overcoming any potential suppression by progesterone in the implant.

While the actions of prolactin on angiogenesis and the vasculature are complicated [review in Ref. (44)] and its causative role in fescue toxicosis is unclear, E_2_ binding to E_2_-α receptors in the plasma membrane of pituitary cells increased prolactin release (45). Estradiol also increased angiogenesis in the pituitary (46), another possible connection for an additive effect of the treatments, with phytoestrogens from SBHs. While elevated serum E_2_ from implants might be relatively quickly metabolized by endogenous enzymes, the clearance rates of phytoestrogen might be slower, resulting in a more sustained physiological response.

Though Carter et al. (7) suggested that SBHs were acting merely through a passive role, diluting the ergot alkaloids, and thereby mitigating their adverse effects, we propose a more active role for SBHs. Both steers with implants or fed SBHs had a reduced percentage of rough hair coat (7). If a decrease in hair coat shedding (another indicator of fescue toxicosis) is a result of decreased blood flow to the hair follicle caused by the ergot alkaloids, then perhaps E_2_ or phytoestrogens/isoflavones can ameliorate the effect by increasing follicular blood flow. Though several reports have found a lack of specific arterial responsiveness to isoflavones [brachial artery (12, 13)], a more global vascular effect was seen, including reduced peak flow velocity and lower peripheral flow resistance. Nestel et al. (47) noted that soy isoflavones increased vascular blood flow in menopausal and perimenopausal women. In addition, using biopsies of subcutaneous fat from men, the phytoestrogen genistein increased relaxation of small arteries, as determined by myograph (14). It is well accepted that arteries often have individual or tissue-specific responses to chemical stimuli, and that global measures, such as blood pressure and velocity, are not necessarily indicated by the responses of individual arteries (48). Though specific direct evidence may be hard to find, isoflavones of SBHs could be ameliorating coat effects via interaction with the vasculature nourishing the hair coat.

Mechanistically, vasorelaxation can be caused by nitric oxide (NO), which is generated by binding and activation of estrogenic receptors by E_2_ or isoflavones (15). While NO was not measured in the current study, Schreihofer et al. (49) measured increased vasorelaxation in ovariectomized rats fed high-soy diets or treated with E_2_. The increase was associated with a decrease in caveolin-1 activity, an enzyme that inhibits NO-induced vasorelaxation. Similar mechanisms could be expected to occur with SBH feeding of steers.

There is a tendency to focus on “the most estrogenic compounds” in SBHs and consider them the “players,” although it is well known that isoflavones undergo degradation by bacteria in the gut/rumen (32, 50). And while some catabolites or conjugates of phytoestrogens are less active, some are more active, depending on the tissue or assay type. For instance, equol, the ruminal catabolite of daidzein (found in SBHs) has greater vasodilatory effects on the basilar artery of rats than its precursor daidzein (51), though they are equipotent on the carotid artery. Equol has been found not only in ruminal digesta of animals fed legumes, but bioavailability was confirmed by its presence in the kidneys and blood (9, 52). Assay and tissue specificity was also a factor in assignment of a “relative potency factor,” as genistein evaluated by E-Screen (breast tissue origin) was ~6 times as potent as daidzein in eliciting MCF-7 cell proliferation (53), with daidzein and equol ~ equivalent, yet the two had different potencies based on E_2_-receptor affinities and transcriptional efficiencies (54).

## Conclusion

The estradiol–progesterone implant and SBH treatments enhanced E_2_Eqs in sera of steers, with an additive effect when combined. It cannot be concluded that the increases in estrogenicity directly caused the mitigation of fescue toxicosis reported by Carter et al. (7) for these steers. But based on research with human subjects, exogenous sources of estradiol and isoflavones may relieve the ergot alkaloid-induced vasoconstriction that persists in livestock grazing toxic endophyte-infected tall fescue. Still unknown is the threshold at which estradiol and or isoflavones can alleviate or mitigate toxicosis. The E-Screen assay was shown to be an effective tool in monitoring serum for estrogenic effects of dietary supplementation with isoflavones or estrogenic implants, though threshold concentrations of estrogenic activity that result in vasculature responses have yet to be established.

## Conflict of Interest Statement

The authors declare that the research was conducted in the absence of any commercial or financial relationships that could be construed as a potential conflict of interest.
